# P2X4 receptors (P2X4Rs) represent a novel target for the development of drugs to prevent and/or treat alcohol use disorders

**DOI:** 10.3389/fnins.2014.00176

**Published:** 2014-06-24

**Authors:** Kelle M. Franklin, Liana Asatryan, Michael W. Jakowec, James R. Trudell, Richard L. Bell, Daryl L. Davies

**Affiliations:** ^1^Department of Psychiatry, Institute of Psychiatric Research, Indiana University School of MedicineIndianapolis, IN, USA; ^2^Titus Family Department of Clinical Pharmacy and Pharmaceutical Economics and Policy, School of Pharmacy, University of Southern CaliforniaLos Angeles, CA, USA; ^3^Department of Neurology, University of Southern CaliforniaLos Angeles, CA, USA; ^4^Beckman Program for Molecular and Genetic Medicine, Department of Anesthesia, Stanford UniversityPalo Alto, CA, USA

**Keywords:** purinergic, P2X, P2X4 receptor, alcoholism, ivermectin, alcohol-preferring, *p2rx4*

## Abstract

Alcohol use disorders (AUDs) have a staggering socioeconomic impact. Few therapeutic options are available, and they are largely inadequate. These shortcomings highlight the urgent need to develop effective medications to prevent and/or treat AUDs. A critical barrier is the lack of information regarding the molecular target(s) by which ethanol (EtOH) exerts its pharmacological activity. This review highlights findings implicating P2X4 receptors (P2X4Rs) as a target for the development of therapeutics to treat AUDs and discusses the use of ivermectin (IVM) as a potential clinical tool for treatment of AUDs. P2XRs are a family of ligand-gated ion channels (LGICs) activated by extracellular ATP. Of the P2XR subtypes, P2X4Rs are expressed the most abundantly in the CNS. Converging evidence suggests that P2X4Rs are involved in the development and progression of AUDs. First, *in vitro* studies report that pharmacologically relevant EtOH concentrations can negatively modulate ATP-activated currents. Second, P2X4Rs in the mesocorticolimbic dopamine system are thought to play a role in synaptic plasticity and are located ideally to modulate brain reward systems. Third, alcohol-preferring (P) rats have lower functional expression of the *p2rx4* gene than alcohol-non-preferring (NP) rats suggesting an inverse relationship between alcohol intake and P2X4R expression. Similarly, whole brain *p2rx4* expression has been shown to relate inversely to innate 24 h alcohol preference across 28 strains of rats. Fourth, mice lacking the *p2rx4* gene drink more EtOH than wildtype controls. Fifth, IVM, a positive modulator of P2X4Rs, antagonizes EtOH-mediated inhibition of P2X4Rs *in vitro* and reduces EtOH intake and preference *in vivo*. These findings suggest that P2X4Rs contribute to EtOH intake. The present review summarizes recent findings focusing on the P2X4R as a molecular target of EtOH action, its role in EtOH drinking behavior and modulation of its activity by IVM as a potential therapy for AUDs.

## Introduction

Alcohol use disorders (AUDs) rank third on the list of preventable causes of morbidity and mortality in the United States, having a major national impact that affects over 18 million people and causes over 100,000 deaths annually (Grant et al., 2004; Johnson, 2010; Bouchery et al., 2011). The economic burden to society for AUDs is in excess of $220 billion/year (Bouchery et al., 2011) which exceeds the costs of other leading preventable causes of death such as cigarette smoking and physical inactivity (Naimi, 2011). To compound this dire scenario, few therapeutic options for AUDs are available, and are largely inadequate (Johnson, 2010). These factors highlight the urgent need for the development of new, effective medications to prevent and/or treat alcohol abuse and dependence (Heilig and Egli, 2006; Johnson et al., 2007; Steensland et al., 2007).

Drug development for AUDs is a relatively young field, compared to other diseases such as cancer, cardiovascular, and psychiatric disorders (Litten et al., 2012). At the present time, the only pharmacotherapeutic agents approved by the FDA for the treatment of AUDs are disulfiram (Antabuse®), naltrexone (Revia® and Vivitrol®), and acamprosate (Campral®), with Vivitrol® being an extended-release injectable formulation of naltrexone (Harris et al., 2010; Litten et al., 2012). These drugs attempt to deter alcohol intake by blocking its metabolism or by targeting the neurochemical and neuropeptide systems that lead to craving and dependence (Gewiss et al., 1991; Colombo et al., 2007; Steensland et al., 2007; Johnson, 2010; Litten et al., 2012). These traditional approaches, even in combination with psychological strategies, have had limited success. The continuing high rates of alcohol abuse coupled with the failure of current treatment strategies have led to considerable effort directed toward the development of new drugs to treat alcohol abuse and dependence (Johnson, 2010; Litten et al., 2012). However, a critical barrier in this endeavor is the lack of information regarding the molecular target(s) by which alcohol exerts its pharmacological activity.

## Ion channels are important targets of ethanol action

Ligand-gated ion channels (LGICs) are widely held to play an important role in ethanol-induced behaviors and drinking (Deitrich et al., 1989; Weight et al., 1992; Dildy-Mayfield et al., 1996; Mihic et al., 1997; Cardoso et al., 1999; Harris, 1999; Woodward, 2000; Davies and Alkana, 2001). Research in this area has focused on investigating the effects of ethanol on two large “superfamilies” of LGICs: (1) The nicotinic acetylcholine receptor superfamily (cys-loop) with members including nicotinic acetylcholine receptors (nAChRs), 5- hydroxytryptamine type 3 receptors (5-HT_3_Rs), γ-aminobutyric acid type-A receptors (GABA_A_Rs) and glycine receptors (GlyRs) (Betz, 1990; Ortells and Lunt, 1995) and (2) the glutamate superfamily (Monaghan et al., 1989; Sommer et al., 1992).

## Overview of purinergic receptors

Within the mammalian CNS, two major purinergic receptor families that bind adenine nucleotides including adenosine 5′-triphosphate (ATP), consisting of the P2X family of LGICs and the P2Y family of G protein-coupled receptors (Burnstock et al., 2011). In addition to binding ATP, the P2Y receptor class also binds adenosine 5′-diphosphate (ADP) as well as uridine nucleosides and nucleotides. A third member of this neurotransmitter class, termed P1, binds adenosine and includes the adenosine receptors A1, A2a, A2b, and A3. Structurally, the P2X and P2Y families are composed of 7 and 8 unique subunits (Coddou et al., 2011), respectively, and are widely expressed in a number of distinct regions within the CNS (Soto et al., 1997).

Thus, purinergic P2X receptors (P2XRs) form functional heteromeric or homomeric receptors (Coddou et al., 2011). Presynaptic P2XRs modulate the release of other neurotransmitters which may be co-released with ATP (Burnstock, 2004; Khakh and North, 2006). Due to the high calcium permeability of these receptors, researchers also have indicated that post-synaptic P2XRs modulate synaptic transmission and interneuronal signaling (Khakh and North, 2006; Jarvis and Khakh, 2009). Since the focus of this review is primarily on the P2X4 receptor (P2X4R) and its role in alcohol drinking behavior, this receptor will be discussed in light of its distribution, expression levels, and physiological roles in modulating neurotransmission in those circuits of the brain known to regulate these behaviors.

While much of our understanding of the P2X4R is derived from its cloning and expression in the rat and mouse (Bo et al., 1995), the human ortholog, termed hP2X4, has been cloned and shares 87% sequence identity with the rat polypeptide (Garcia-Guzman et al., 1997). Structurally, the P2X4R channel is arranged as a trimeric complex at the cell membrane and functions as a LGIC for both monovalent (Na^+^ and K^+^) and divalent (Ca^2+^) cations. In fact, at resting potentials, P2X4Rs are able to flux high Ca^2+^ concentrations, at levels approaching or exceeding those of the N-methyl-_D_-aspartate (NMDA) subtype of glutamate receptor (Abbracchio et al., 2009). Since the NMDA receptor is blocked by Mg^2+^ ions at resting potentials, P2X4Rs as well as other P2XRs, may play an important role in synaptic plasticity potentially complementing the regulatory role of the α-Amino-3-hydroxy-5-methyl-4-isoxazolepropionic acid (AMPA) subtype of glutamate receptor (Kessels and Malinow, 2009). Several reports suggest that P2X4Rs contribute to the induction of hippocampal long-term potentiation (LTP), and possibly long-term depression (LTD), directly influencing synaptic strength (Cunha et al., 1996; Cunha and Ribeiro, 2000; Yamazaki et al., 2003; Fujii, 2004). For example, mice lacking the *p2rx4* gene (P2X4-KO) displayed reduced induction of LTP (Sim et al., 2006). Therefore, P2X4Rs themselves may play a role in maintaining homeostatic plasticity and influencing the threshold for LTP and LTD induction (Pankratov et al., 2002).

Both *in situ* hybridization histochemistry and immunohistochemical staining [light and electron microscopy (EM) immunogold labeling] in conjunction with western immunoblotting have demonstrated that P2XRs are distributed widely within the mammalian CNS in a heterogeneous fashion. P2X4Rs, along with P2X2Rs and P2X6Rs, are the most robustly expressed functional ATP-gated purinergic receptors in the CNS (e.g., Khakh, 2001; Amadio et al., 2007; Surprenant and North, 2009; Coddou et al., 2011), and are found on most neurons and glial cells (Burnstock and Knight, 2004; Ulmann et al., 2008; Choi et al., 2009). The P2X4Rs are localized in the hippocampal pyramidal layers CA1, CA2, and CA3 as well as the granule layers of the dentate gyrus, Purkinje cells of the cerebellum, the cerebral cortex including both somatosensory and prefrontal regions, the medial habenula, olfactory bulb, and spinal cord (Bo et al., 1995; Buell et al., 1996; Kanjhan et al., 1999; Rubio and Soto, 2001). Analysis with EM immunostaining suggests that P2X4Rs are preferentially localized to the post-synaptic terminal, predominantly toward the periphery of the synapse (Rubio and Soto, 2001). However, additional studies have suggested that P2X4Rs are localized to both the pre-synaptic as well as post-synaptic terminal in several brain regions (Rubio and Soto, 2001; Burnstock et al., 2011). Based on physiological studies, P2X4Rs can form homo-multimeric complexes with themselves or hetero-multimeric assemblies with P2X6, for example (Torres et al., 1999). Recent studies mapping P2X4R expression have now identified co-localization of these receptors within dopaminergic neurons within the substantia nigra pars compacta (SNpc) as well as GABAergic neurons in both the striatum and substantia nigra pars reticulate (SNpr) (Amadio et al., 2007). Administration of ATP to the dopaminergic neurons of the SNpc can modulate firing activity as well as the release of presynaptic dopamine (Choi et al., 2009). Similar regulation of dopamine release in the nucleus accumbens (NAc) has also been reported (Krugel et al., 2003). In addition to normal levels of expression, the P2X4R has been reported to be altered by dopamine depletion, specifically the 6-hydroxydopamine rat model of Parkinson's disease, resulting in decreased expression in the SNpc and dorsal striatal GABAergic neurons, and increased expression in SNpr GABAergic neurons (Amadio et al., 2007). Taken together these findings support a close relationship between P2X4R expression and dopamine neurotransmission.

**Figure 1:**
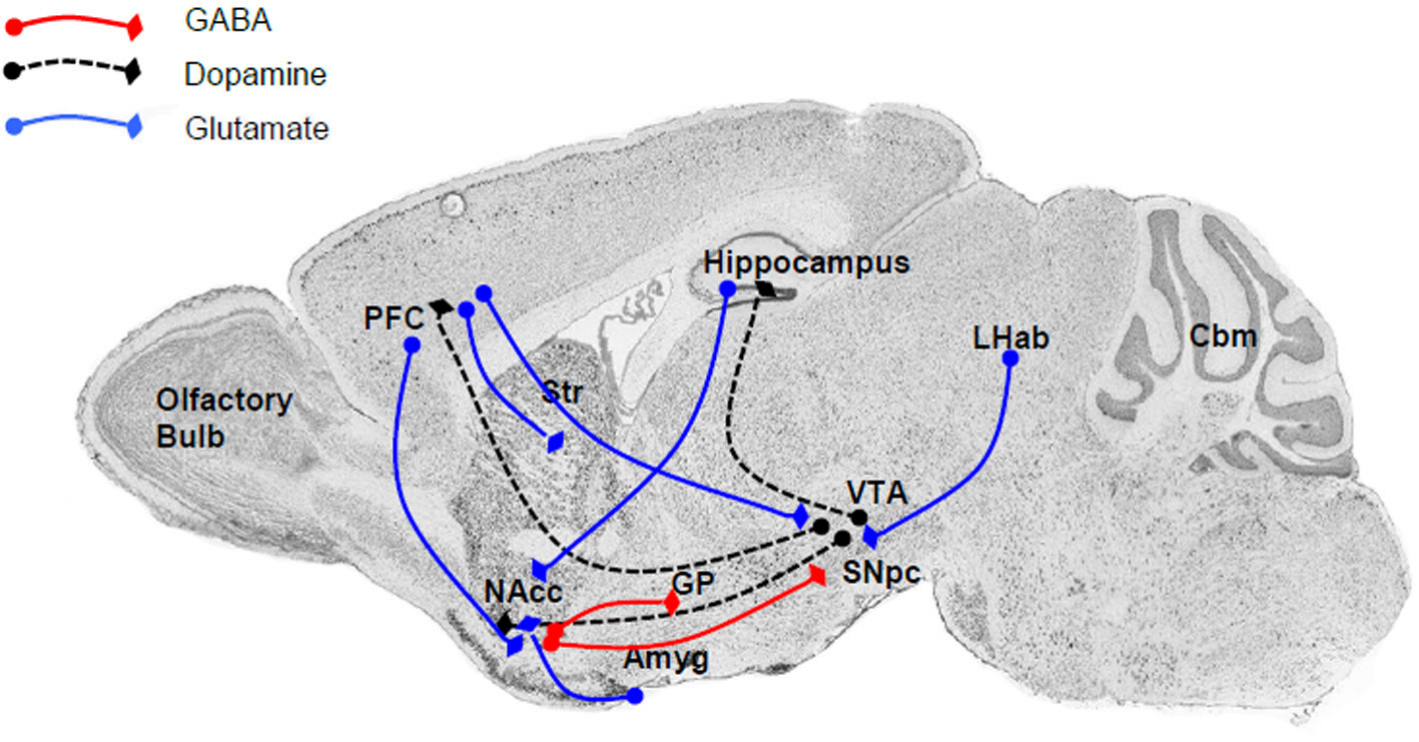
**The major connections within the reward and aversion circuitry are regulated by P2X4Rs either directly or indirectly**. Within these neuroanatomical regions P2X4Rs either colocalized to neuronal cell bodies or localized to glia and modulate the release of various neurotransmitters. Major neurotransmitters and their pathways include (i) dopamine (black dash) linking the VTA with the NAcc (ventral striatum), PFC, and hippocampus; (ii) glutamate (blue line), an important neurotransmitter linking the PFC to the NAcc, striatum, and VTA, as well as between the LHab and VTA, hippocampus and NAcc, and Amyg and NAcc; and GABA (red line) between the NAcc and SNpc or GP. Abbreviation: Amyg, amygdala; Cbm, cerebellum; GP, globus pallidus; LHab, lateral habenular; NAcc, nucleus accumbens; PFC, prefrontal cortex; SN, substantia nigra; Str, striatum; VTA, ventral tegmental area.

The close relationship between the P2X4R and alcohol drinking behavior is supported by the localization of P2X4Rs within the reward circuitry of the mammalian brain (Figure 1), specifically the mesolimbic and mesocortical pathways that consist of connections originating in the ventral tegmental area (VTA) of the midbrain, connecting to the NAcc (also termed the ventral striatum), amygdala, limbic system, hippocampus, and prefrontal cortex (Gonzales et al., 2004). Within this circuitry, P2X4Rs have been localized to these regions either within neurons (Rubio and Soto, 2001) or glia (both microglia and astrocytes) (Rubio and Soto, 2001; Ulmann et al., 2008, 2013), implicating a role in reward- and aversion-related neurotransmission, as well as cellular immune activity. Functional studies also have reported that P2X4Rs can play an important role in the reward circuitry by regulating the release of glutamate (Krugel et al., 2004; Khakh, 2009) or dopamine (Krugel et al., 2003; Kittner et al., 2004) within the VTA and NAcc. In addition, the identification of ATP as a co-neurotransmitter in both glutamatergic and dopaminergic neurons strongly implicates the role of P2X4Rs and other purinergic receptor signaling in synaptic transmission as well as synaptic plasticity within these regions (Krugel et al., 2003).

Neuronal and glial P2X4Rs and other P2XRs regulate neurotransmission directly. They also may be involved in a number of distinct mechanisms influencing synaptic plasticity within the reward circuitry, thus influencing alcohol-drinking behaviors. For example, the expression and utilization of neurotrophic factors such as brain-derived neurotrophic factor (BDNF) (Trang et al., 2009; Klein et al., 2012) and nerve growth factor (NGF) (D'Ambrosi et al., 2001) can modulate synaptic plasticity by modulating LTP induction. It is well-accepted that neuro-inflammatory processes play both deleterious and beneficial roles in synaptic plasticity and cell death (Wee, 2010). The fact that P2X4Rs are localized in microglia suggests that these receptors may regulate the activation, migration, cytokine release, and physiological roles of microglia at sites of neuroplasticity, including injury (Tsuda et al., 2003; Sim et al., 2007). It is not yet known if these regulatory processes through microglia lead to the loss of synaptic connectivity or play a regulatory role to enhance synaptic strength. ATP levels also can be regulated enzymatically through expression of ecto-nucleotidases thus influencing purinergic receptor activity such as seen in the hippocampus in models of learning (Bonan et al., 2000; Rucker et al., 2004). Finally, P2X4Rs have been reported to modulate signaling in a wide spectrum of LGICs including those receptors for GABA_A_, glycine, nicotinic acetylcholine, and glutamate demonstrating its wide and complex sphere of influence (Adelsberger et al., 2000; Andries et al., 2007; Sattelle et al., 2009; Tabakoff et al., 2009; Baxter et al., 2011).

Activation of these LGIC receptors has been reported to mediate some of ethanol's behavioral effects (Grant, 1994). The modulatory interaction of P2X4Rs with these other ethanol-related receptor families likely is indicative of an indirect association between P2X4Rs and some of the molecular actions of ethanol. In addition, growing evidence supports a more direct interaction between P2X4Rs and the effects of ethanol. Alcohol drinking and abuse behaviors share many similarities with addictive behaviors to drugs of abuse such as cocaine. Purinergic receptors such as P2X4Rs are in a position to influence reward and aversive circuits especially those involving dopamine thus implicating this class of receptors in modulation of synaptic signaling associated with addictions. Therefore, the P2X4R may serve as an important therapeutic target in treating alcohol abuse behaviors (Davies et al., 2005; Asatryan et al., 2011; Litten et al., 2012). This line of research is further supported by the direct demonstration of interactions with ethanol on P2X4Rs and the fact that drugs that potentiate P2X4R neurotransmission such as IVM can reverse some of ethanol's behavioral effects (Davies et al., 2005; Ostrovskaya et al., 2011).

## Ethanol inhibits the function of P2X4Rs

To date, P2X4Rs are the most ethanol-sensitive P2XR subtype tested. Findings indicate that intoxicating and anesthetic ethanol concentrations desensitize the response of P2X4R-expressing cell lines to ATP exposure (Li et al., 1993; Xiong et al., 2000; Davies et al., 2002, 2005; Ostrovskaya et al., 2011), altering the ability of these receptors to modulate neurotransmission. Initial voltage clamp studies in the late 1990's, testing rat recombinant homomeric P2X4Rs expressed in *Xenopus* oocytes found that ethanol caused concentration-dependent inhibition of ATP-induced currents with the IC_50_ determined at 58 mM (Xiong et al., 2000). Caution must be taken when interpreting the IC_50_ value in this study (Xiong et al., 2000) as it was determined by testing a range of ethanol concentrations from 1 to 500 mM. Later studies using two-electrode voltage clamp confirmed the inhibitory effects of ethanol in rat P2X4Rs, but focused on lower concentrations (25–200 mM) (Davies et al., 2002, 2005; Asatryan et al., 2010). The extent of ethanol inhibition was 50–60% at 200 mM. Notably, the inhibitory effects of ethanol on ATP-gated P2X4R function were not dependent on the expression or recording system as similar results were found when tested in frog oocytes, transfected HEK293 cells, and lentivirally transduced mouse hippocampal neurons (Ostrovskaya et al., 2011). Ethanol-induced P2X4R inhibition may provide some insight regarding the role of P2X4Rs in ethanol intake (described below) (Li et al., 1998; Weight et al., 1999; Xiong et al., 2000; Davies et al., 2005).

## The P2X4R mechanism of ethanol inhibition

Recent investigations from two-electrode voltage clamp and patch clamp studies demonstrate that ethanol inhibition of ATP-activated currents in P2X4Rs is not dependent on membrane potential (from −60 to +20 mV), and ethanol does not change the reversal potential of ATP-activated current (Xiong et al., 2005; Ostrovskaya et al., 2011). This work argues that ethanol does not affect the receptor function directly, but rather act as a negative allosteric modulator as illustrated by its ability to shift the ATP concentration response curve to the right and increase the EC_50_ value significantly (Davies et al., 2002, 2005).

Earlier investigations suggested that ethanol might inhibit P2X4Rs by decreasing the apparent affinity of the binding site for ATP (Xiong et al., 2005). However, recent studies using patch-clamp and a HEK 293 expression system provided more insights into the intricate mechanisms of ethanol action in P2X4Rs (Ostrovskaya et al., 2011). In this latter study, the authors reported that there was no difference in the magnitude of ethanol inhibition at a wide range of ATP concentrations, which contradicted the previous findings that a more prominent effect was present at the lower end of the tested ethanol concentrations (Ostrovskaya et al., 2011). The observation that there was no change in channel deactivation supported the recent findings. These studies also demonstrated that ethanol interaction with P2X4Rs is rapid with very fast rates of association and dissociation (Ostrovskaya et al., 2011). Moreover, the findings suggested a use-dependent mechanism for the action of ethanol consistent with properties of an open-channel blocker. Interestingly, in contrast to classic open-channel blockers, ethanol did not stabilize the open/desensitized states of the channel and did not change the ion permeability ratio. These properties are consistent with a low-affinity modulator “binding,” which is a dynamic series of on and off bounces in the putative binding sites or interaction pockets.

## Sites of ethanol action in P2X4Rs

Knowledge regarding the molecular targets of ethanol in P2X4Rs remains limited. Site directed mutagenesis as well as chimeric construction strategies have been used to look into the important molecular sites for the action of ethanol in P2XRs. Earlier work from the Davies lab that used a chimeric construction approach between P2X2 and P2X3 receptor subunits found that the interface between the transmembrane (TM) segments and the ectodomain, as well as the TM segments themselves, are important for the actions of ethanol (Asatryan et al., 2008). These studies were extended to P2X4Rs where an alanine scan of the indicated regions was performed via site-directed mutagenesis (Popova et al., 2010). The studies identified two residues at the ectodomain-TM2 interface, i.e., Asp331 and Met336, that when mutated to alanine significantly reduced ethanol inhibition of ATP-gated currents (Popova et al., 2010). Interestingly, Asp331 mutations affected ethanol sensitivity at higher ethanol concentrations (>50 mM), whereas Met336 mutations were sensitive to lower, intoxicating ethanol concentrations (10 mM). These mutations did not cause significant changes in the agonist response, i.e., ATP Imax, EC_50_, or Hill slope. More detailed study of the physico-chemical properties of these identified sites using the mutagenesis approach and correlation analyses revealed a significant relationship between the ethanol response and hydropathy as well as polarity, but not the molecular volume/molecular weight of the residues at these two positions (Popova et al., 2010). Further patch clamp studies focusing on the identified residues confirmed that they are involved in ethanol inhibition (Ostrovskaya et al., 2011). In addition, these studies suggested that Met336 and Asp331 play a role in P2X4R gating.

The involvement of Asp331, Met336 in an action pocket for ethanol was confirmed later on the first molecular model of the rat P2X4R (Asatryan et al., 2010). This model also demonstrated that Trp46 in the TM1 segment of the receptor contributed to the formation of the putative binding pocket. The important role of Trp46 in these molecular interactions was later supported experimentally (Popova et al., 2013). Alanine substitution at position 46, but not at any other positions of the TM1 segment, abolished ethanol inhibition of P2X4Rs (Popova et al., 2013). Importantly, the presence of an aromatic ring at position 46 appeared to be required for the maximal response to ethanol. Notably, as with Asp331 the reduction of ethanol inhibition was found at higher ethanol concentrations (≥50 mM). These findings are relevant to chronic alcohol abusers whose blood ethanol concentrations generally reach levels as high as 50 mM and above. The findings also supported the notion that there are multiple sites for ethanol action in P2X4Rs—(1) high affinity sites (such as position 336) that are sensitive to lower intoxicating concentrations experienced by most social drinkers and (2) low affinity sites (such as positions 46 and 331) that display responses at the higher concentrations achieved by chronic alcoholics. Taken together, these findings suggest that there is a direct interaction of ethanol with P2X4Rs, which may have important implications for the role of these receptors in AUDs. This suggestion is supported by P2X4R populations in central areas that promote alcohol drinking.

## Current *in vivo* support for involvement of P2X4Rs in alcohol drinking

As presented above, P2XRs have been associated regionally and functionally with several CNS areas implicated in with ethanol consumption and reinforcement (Krugel et al., 2003; Amadio et al., 2007; Heine et al., 2007). For example, evidence suggests that dense P2X4R populations on medium spiny neurons, interneurons, and microglia in the VTA may modulate ethanol-induced changes to GABA and glutamate neurotransmission and dopamine levels in the NAcc (Xiao et al., 2008). These areas are highly related to the regulation and reinforcing effects of ethanol and other drugs of abuse (Pierce and Kumaresan, 2006) and disruptions in these areas may contribute to the development of AUDs. Moreover, the high expression of P2X4Rs in these areas may suggest that these receptors are involved in the some of the behavioral, cellular, and molecular effects of ethanol (Pankratov et al., 2009; Tabakoff et al., 2009).

In addition, regional differences in P2X4R levels may be involved in innate alcohol preference. Several research groups have used gene expression profiling to demonstrate differences in *p2rx4* mRNA in animals genetically selected for divergent ethanol intake. First, Kimpel et al. (2007) reported that *p2rx4* mRNA was reduced in composite preparations of five brain regions from inbred alcohol-preferring (iP) rats, relative to their non-preferring (iNP) counterparts. Second, reduced *p2rx4* levels were found in the VTA of male alcohol-preferring (P) vs. -non-preferring (NP) and high alcohol drinking vs. low alcohol drinking (HAD1/LAD1) rats (McBride et al., 2012). Interestingly, these researchers reported that *p2rx4* expression was increased in a replicate high-alcohol drinking (HAD2) rat line, relative to its low-alcohol drinking (LAD2) counterparts (McBride et al., 2012). Taken together, evidence suggests that disparate *p2rx4* gene expression contributes to high alcohol drinking phenotypes. Further, these findings highlight the significance of genetic heterogeneity in developing medications for the treatment of AUDs and emphasize the importance that pharmacogenetics likely will play in future medications development (Johnson, 2010; Heilig et al., 2011).

The above studies were conducted in ethanol-naïve subjects with a genetic propensity to consume large quantities of alcohol. In line with these findings, growing evidence from pre-clinical animal models suggests that functional differences in P2X4Rs manifest behaviorally in altered alcohol drinking levels. Tabakoff et al. (2009) found that lower levels of whole brain expression of *p2rx4* mRNA in inbred rats were associated with a high ethanol-drinking phenotype compared to those with a lower ethanol-drinking phenotype. Simply put, expression of *p2rx4* was negatively correlated with 24-h two-bottle free choice alcohol intake. In agreement with this finding, male P2X4-KO mice displayed increases in 10% ethanol intake under intermittent 4-h limited access conditions, and under 24-h access conditions, relative to wild-type (WT) mice (Wyatt et al., 2014). Furthermore, in the same report (Wyatt et al., 2014), it was found that acquisition of 24-h, two-bottle choice alcohol (vs. water) drinking occurred earlier in male P2X4-KO mice, compared to WT controls. As P2X4R-KO mice display differences in ethanol intake and some of the sedative-hypnotic properties of ethanol, as well as exhibiting elevations in GABA_A_R expression in the cerebellum, relative to WT mice (Wyatt et al., 2014), it is possible that one role for P2X4Rs is to regulate some of ethanol's GABA-mediated behavioral effects.

This suggestion is in line with the ability of P2X4Rs to modulate GABA neurotransmission and may indicate that these receptors are involved in GABA-mediated regulation of ethanol consumption (Rewal et al., 2009). However, it has been difficult to develop an efficacious clinical assessment for the direct involvement of P2X4Rs in the human alcoholic condition. Therefore, selective pharmacological agents that target P2X4Rs have been utilized in several pre-clinical laboratory studies to establish their ability to affect ethanol intake. Ivermectin (IVM), in particular, is being investigated for its ability to reduce ethanol intake in pre-clinical studies and in a developing clinical trial, due to its selective modulation of P2X4Rs within the P2XR superfamily.

## IVM as a potential clinical tool for treatment of AUDs; insights from P2X4Rs

IVM is an FDA-approved semi-synthetic macrocyclic lactone avermectin used in veterinary and clinical medicine (Richard-Lenoble et al., 2003; Geary, 2005; Fox, 2006; Gonzalez et al., 2012). As an anti-parasitic agent, IVM has been administered to millions of humans, with few reports of severe adverse side effects (Guzzo et al., 2002; Omura, 2008). Its positive safety profile has been echoed in clinical reports at doses up to 10 times the recommended levels (Guzzo et al., 2002).

IVM's utility as an antihelminthic agent generally is attributed to its ability to potentiate non-mammalian glutamate-gated inhibitory chloride channels (Cully et al., 1994; Dent et al., 1997). In mammals, IVM acts at a number of ion channels including GABA_A_Rs and GlyRs (for review see Dawson et al., 2000; Shan et al., 2001) and this action is thought to produce anticonvulsant effects in mice (Dawson et al., 2000). Despite robust findings that IVM enhances inhibitory neurotransmission and decreases excitatory transmission (e.g., Shan et al., 2001; Ikeda, 2003), evidence suggests that IVM blocks some of the behavioral effects of GABA agonists (Davies et al., 2013). IVM also has been found to act as a selective, positive allosteric modulator of ATP-evoked currents at P2X4Rs (Khakh et al., 1999; Priel and Silberberg, 2004; Lalo et al., 2007). Further sections of this review present pharmacological and pre-clinical evidence suggesting that IVM may serve as a useful clinical tool for the treatment of AUDs.

## IVM antagonizes the inhibitory effects of ethanol on P2X4Rs by acting on common sites in P2X4Rs

Previous reports suggested that IVM binds at the lipid–protein interface, acting at sites located within the TM segment and at the ectodomain-TM interface of the P2X4R (Jelinkova et al., 2006, 2008; Silberberg et al., 2007). Based on the similar structural loci for ethanol and IVM action in P2X4Rs, later studies were performed to test whether IVM would interfere and reduce the effect of ethanol on P2X4Rs. Using a *Xenopus* oocyte expression system and two-electrode voltage clamp, these studies were the first to demonstrate that IVM antagonized ethanol effects in P2X4Rs in a concentration-dependent manner (Asatryan et al., 2010). Importantly, 0.5 μM IVM eliminated the inhibitory effect of pharmacologically relevant concentrations of ethanol (25, 50 mM) without evidence of causing potentiation in P2X4Rs. In addition, these studies revealed that the interaction is competitive in nature; suggesting that the drugs have a common molecular target/site(s) in P2X4Rs. Consistent with the latter notion, further studies found that Met336 at the ectodomain-TM2 interface and Trp46 in the TM1 segment played a role in the action of both drugs. Mutations at position 336, which were known to alter the sensitivity of P2X4Rs to ethanol (Popova et al., 2010), also altered the receptor's sensitivity to IVM (Asatryan et al., 2010; Popova et al., 2013). Moreover, the physico-chemical requirements for the residue at this site were similar for the actions of both drugs. Specifically, the hydrophobic side chain of Met336 played a critical role in governing the actions of ethanol and IVM. At position 46, substitution of non-aromatic residues reduced the effect caused by ethanol concentrations that were greater than 50 mM and shifted IVM response from potentiation to inhibition (Popova et al., 2013). Furthermore, the findings from the double mutant W46A–M336A P2X4Rs suggested an important role of the Trp46 and Met336 interaction for ethanol and IVM action. Similar to the effects in the single mutants (W46A and M336A), there was: (1) reduced response to ethanol and (2) a shift of the IVM response similar to the effect found with non-aromatic mutants at position 46. Collectively, the findings supported the notions that positions 46 and 336 are common sites for the actions of IVM, serving as sites for the antagonistic effect of IVM. Importantly, the interaction between residues at these positions contributes to the putative binding pocket for both drugs. The presence of this pocket was supported by molecular modeling studies.

## Molecular modeling reveals a putative binding pocket for ethanol and IVM in P2X4Rs

**Figure 2:**
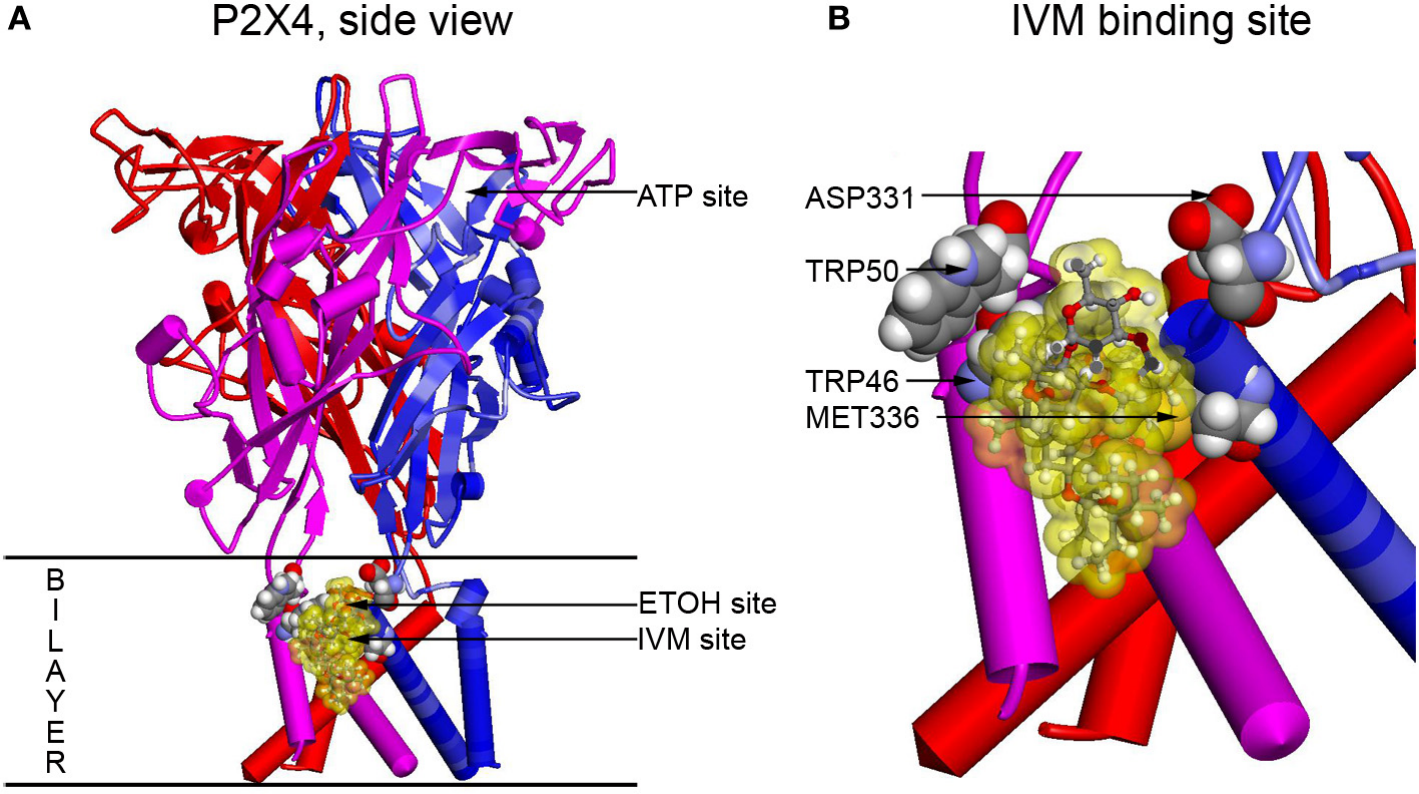
**Full size and zoomed models of the rat P2X4R**. A homology model of rat P2X4R was built by threading the rat primary sequence on the zebrafish X-ray structure in the open conformation (PDB ID 4DW1) (Hattori and Gouaux, 2012), essentially as described before (Popova et al., 2013). **(A)** A side view of the full P2X4R as viewed in the plane of the membrane looking toward the ion pore from the outside. Horizontal lines indicate the predicted extent of the membrane bilayer. The ATP binding site is over 20 Angstroms distant from the IVM binding site, yet there is substantial interaction between them. Residues important for IVM binding are rendered with space filling surfaces (carbon, oxygen, nitrogen, and hydrogen are colored gray, red, blue, and white). IVM is rendered in ball and stick and surrounded by a transparent yellow surface to outline possible interactions with the protein. A putative EtOH binding site, based on the mutations, is indicated by an arrow (EtOH site). **(B)** The IVM binding site is zoomed to reveal details about interactions with residues identified as important by mutagenesis. Abbreviation: P2X4R, purinergic P2X4 receptor; ATP, adenosine 5′-triphosphate; IVM, ivermectin; EtOH, ethanol.

To visualize residues experimentally found to be important for ethanol and IVM action (Met336 and Trp46) and verify possible interactions between these and other residues, molecular modeling approach was applied. The first homology model of rat P2X4 was built using as a template the zebrafish P2X4 3.1 Å resolution crystal structure in the closed state published in 2009 by the Gouaux group (Kawate et al., 2009). This model revealed a pocket formed by Asp331, Met336, Trp46, and Trp50 that was suggested to play a role in the actions of ethanol and IVM. It was demonstrated that the Trp46 and Trp50 side chains of the first alpha helix face Asp331 and Met336 in the final alpha-helix of the adjacent subunit (Figure 2). A site was formed by manual rotations of the C-alpha to C-beta bonds of Trp46 and Trp50 in which the two Trp side chains and the sulfur of Met336 interact. An ethanol molecule was fitted manually in the site while considering a combination of cation-Pi interactions between the hydrogen of ethanol and the tryptophans and additional interactions between the methionine sulfur atom and the oxygen atom of ethanol. This first model of P2X4Rs revealed that several amino acid residues known to be important for the effects of ethanol on these receptors, although seemingly widely scattered in the primary sequence of P2X4R, were actually clustered around a small cavity in the three-dimensional structure.

The molecular model was revised later in the light of the new open state crystal structure of the zebrafish P2X4R (Hattori and Gouaux, 2012) and new findings on ethanol and IVM binding (Popova et al., 2013). It provided insights into the role of the molecular interactions in the responses to ethanol and IVM. The model supported the previous one confirming that there is a binding pocket in P2X4Rs formed by positions 46, 331, and 336. In addition, position 42 was added to the residues that play a role in the formation of the pocket and further experimental work supported that finding. This model also confirmed that Trp46 and Met336 face each other (Asatryan et al., 2010). It was proposed that interactions between the aromatic rings and sulfur groups may occur in number of ways; including hydrogen bonding to the aryl hydrogens, SH–Pi interactions, electrostatic, or hydrophobic interactions (Tatko and Waters, 2004). Importantly, interactions of the sulfur atom of methionine with multiple aromatic groups can produce “hot spots” in protein–protein interactions (Ma and Nussinov, 2007) contributing to the stabilization of the protein (Tatko and Waters, 2004).

Aromatic residues located within the upper part of the TM1 segment of P2X4Rs, such as Tyr42, Trp46 and Trp50, have been suggested to play a role in the three-dimensional organization of the receptor through Pi-Pi interactions (Jelinkova et al., 2008). The model in Figure 2 demonstrated that Trp46 is “sandwiched” between Tyr42 and Trp50 (Popova et al., 2013). Interestingly, disruption of the Pi-Pi interactions between Trp46 and Tyr42 but not Trp46 and Trp50 seemed to alter ethanol and IVM responses. Formation of the hydrogen bond between the phenolic hydroxyl group of Tyr 42 and hydroxyl oxygen of IVM also were deemed to be important for IVM binding. Substituting Tyr42 for Phe, which lacks the hydroxyl group on the aromatic ring, significantly altered ethanol and IVM responses (Popova et al., 2013). These findings suggest the importance of Tyr42 in the modulation induced by the two drugs.

The new model also provided insights into the binding of the IVM molecule in the identified putative binding site. The model replicated the vertical and lateral positions of IVM in the X-ray structure of the pentameric glutamate-gated chloride ion channel GluCl (Hibbs and Gouaux, 2011). The van der Waals surface of IVM molecule fit well within one of the 39 cavities found in P2X4R model. Molecular dynamic simulations demonstrated that the IVM molecule was stable in that position. The sites identified thus far as important for the action of IVM in P2X4Rs are located close to fenestrations that were described recently at the membrane interface of P2X4Rs (Samways et al., 2012; Jiang et al., 2013). In the open state of the receptor, these “lateral portals” open to approximately 8 Å (Jiang et al., 2013) which is sufficient for the IVM binding. Consistent with the latter, IVM is known to bind and stabilize the open state conformation of the receptor.

Taken together, the molecular models built on the closed and open state zebrafish P2X4R structure suggested for the first time the presence of nearly co-localized sites in an ethanol and IVM action pocket in P2X4Rs formed by amino acid residues at positions 42, 46, 331, and 336 (Asatryan et al., 2010). This action pocket represents a potential target for development of medication to treat AUDs. Moreover, growing evidence suggests that the interactions of ethanol and IVM apparent in molecular models may be indicative of IVM's efficacy to regulate ethanol-related behaviors, including consumption.

## Behavioral pharmacological evidence for the anti-ethanol effects of IVM

In addition to direct actions at P2X4Rs and in line with some P2X4R functions, IVM likely affects several neural signaling systems that are modulated by P2X4Rs and have been reported to be involved in alcohol drinking, such as GABA, glutamate, and dopamine (Xiao et al., 2008; Vavra et al., 2011). Further, IVM independently affects several neural systems associated with alcohol drinking. For example, IVM potentiates activation of GlyRs (Lynagh et al., 2011; Wang and Lynch, 2012), which have been identified as one of a few known primary targets for alcohol (cf. Vengeliene et al., 2008). In addition, IVM enhances α_7_ nAChR function (cf. Daly, 2005), which is pertinent due to the inhibiting actions of alcohol on these receptor subtypes (cf. Davis and de Fiebre, 2006).

Converging pre-clinical evidence suggests that a single systemic IVM exposure reduces alcohol intake and reinforcement under binge-like and 24-h choice drinking conditions in male and female mice and during a 3-h operant session in male rats (Kosten, 2011; Yardley et al., 2012; Asatryan et al., 2014). These studies suggest that acute IVM administration is effective at reducing ethanol intake, despite its low CNS accumulation (Yardley et al., 2012; Asatryan et al., 2014). In addition, this work found that IVM-induced reductions in ethanol intake were proportional to detectable central IVM levels (Yardley et al., 2012). The estimated times to maximal CNS IVM levels have ranged from 2–5 (Borst and Schinkel, 1996) to 8–10 h (Yardley et al., 2012; Asatryan et al., 2014). This low CNS accumulation is coupled with a lengthy clearance period (plasma half-life: 12.67 h; brain half-life: 19 h) (Asatryan et al., 2014). Interestingly, Yardley et al. (2012) reported that 7-day repeated peripheral administration with a sub-threshold (1.25 mg/kg) dose of IVM reduced ethanol intake in C57BL/6 mice under sub-chronic 24-h continuous access conditions. This additive effect of dosing likely is related to the slow clearance of the medication from the system.

The LD(50) for IVM has been reported to range from approximately 18 to 50 mg/kg depending upon the subjects' sex and species (Dadarkar et al., 2007; Trailovic and Varagic, 2007), which far exceeds the reported therapeutic dose levels for reduction of ethanol intake and reinforcement (2.5–10 mg/kg) (Kosten, 2011; Yardley et al., 2012; Asatryan et al., 2014). However, doses as low as 10 mg/kg have been reported to induce CNS toxicity (Lerchner et al., 2007). Despite these reports, evidence suggests that IVM is well-tolerated, and elicits few adverse effects (Davis et al., 1999; Omura, 2008; Sun et al., 2010) in the absence of a P-glycoprotein (P-gp) deficiency (Lankas et al., 1997).

As a result of IVM's pharmacokinetic characteristics, it is possible that some effects of the compound reflect locomotor alterations. Free-choice oral IVM self-administration may stimulate activity at low concentrations in some mouse strains (Davis et al., 1999). In contrast, i.p. IVM was reported to depress locomotor behaviors in rats, with a threshold dose between 0.5 and 1.0 mg/kg (Spinosa et al., 2002). Systemic IVM injections have been reported to induce motor deficits, such as somnolence and ataxia, although these effects are largely dose-dependent (Spinosa et al., 2002; Dadarkar et al., 2007; Trailovic and Varagic, 2007; Kosten, 2011; Trailovic et al., 2011). Importantly, evidence suggests that co-administration of alcoholic beverages with IVM (Shu et al., 2000) or the use of ethanol as a dissolution medium for IVM (Edwards et al., 1988) increases the bioavailability of the compound, possibly resulting in potentiated locomotor disruptions (Shu et al., 2000). Thus, the viable therapeutic dose window for IVM may require reassessment when administering the compound to alcohol-drinking individuals.

## Involvement of P2X4Rs in the ethanol antagonizing (or anti-ethanol) effects of IVM

Accumulating evidence suggests that IVM may represent a pharmacological tool to investigate the role of P2X4Rs in alcohol drinking. Studies from P2X4-KO mice provided some insights into the role of P2X4Rs in IVM's anti-ethanol effects *in vivo*. These studies found a significantly smaller potential of IVM to reduce ethanol intake and ethanol preference in P2X4-KO vs. WT mice using a 24-h two-bottle choice drinking paradigm (Wyatt et al., 2014). This finding, in conjunction with previous IVM measures (Bortolato et al., 2013) suggests that a significant portion of IVM's ability to reduce alcohol intake is linked to P2X4Rs (Wyatt et al., 2014). The reduced ability of IVM to inhibit ethanol consumption in P2X4-KO mice may also be linked, in part, to changes in the compensatory alterations in the expression profile of LGICs functionally associated with the regulation of ethanol behaviors. Consistent with this notion, the work found significant changes in the expression of α1 subunit of GABA_A_Rs in several brain regions of P2X4-KO mice (Wyatt et al., 2014).

## Using IVM as a platform to develop more efficient medications for alcohol use disorders

As described above, pre-clinical evidence has demonstrated the ability of IVM to reduce ethanol consumption. However, this effect is somewhat limited in magnitude (e.g., Yardley et al., 2012). Despite its lipophilic nature, IVM does not readily achieve high brain concentration, most likely because it is a good substrate for the P-gp transporter. At the present time, there is an ongoing effort to identify and/or develop new compounds that enhances IVM's retention in the CNS while maintaining its ability to reduce alcohol intake and maintain its high tolerability and broad safety profile. It is thought that modification of the IVM structure to reduce its P-gp substrate recognition (Lespine et al., 2007; Menez et al., 2012) and alteration of its interaction with a targeted brain receptor should positively impact the drug's ability to reduce ethanol intake (Asatryan et al., 2014). Support for this notion comes from recent work comparing the effects of IVM with two IVM-related macrocyclic lactones, abamectin (ABM) and selamectin (SEL), for their abilities to cross blood brain barrier (BBB); reduce ethanol intake in mice; and to alter modulation of GABA_A_Rs and P2X4Rs expressed in *Xenopus* oocytes (Asatryan et al., 2014). Interestingly, SEL, despite having a 50X plus higher concentration in the brain (compared to IVM), displayed minimal ability to reduce ethanol intake in mice in a 24 h two-bottle choice paradigm and was minimally effective in antagonizing the effects of ethanol in recombinant P2X4Rs (Asatryan et al., 2014). ABM's brain retention was significantly increased compared to IVM; however, its effects on alcohol intake were comparable to IVM. All three compounds were effective in modulating GABA_A_Rs. Collectively, this work suggested that chemical structure and effects on receptor function are important for the ability of avermectins to reduce ethanol intake and that these factors are more important than BBB penetration alone (Asatryan et al., 2014).

Taken together, these findings support the hypothesis that avermectins can be used as a platform for developing novel drugs to prevent and/or treat AUDs. The findings also suggest that key structure-activity relationships can be used to improve the ability of avermectins to cross the blood brain barrier, while maintaining their effects on P2X4Rs, thus improving their ability to reduce ethanol intake.

## Conclusions

P2X4Rs represent a novel and largely unexplored target for drug development to prevent and/or treat AUDs. This hypothesis stems from a large body of evidence indicating that P2X4Rs play a role in modulation and/or regulation of ethanol intake and that there is an inverse relationship between P2X4R activity and ethanol consumption. Further support for this hypothesis can be taken from the recent finding that IVM, a positive modulator of P2X4Rs, antagonizes ethanol-mediated inhibition of P2X4Rs *in vitro* and reduces ethanol intake and operant ethanol self-administration in rodents. Notably, the findings, to date, support developing novel therapeutic agents for AUDs that focus on this largely unexplored target. To support this endeavor, ongoing and future studies will continue assessing the role of P2X4Rs in reward systems linked to alcohol intake, which should provide new insights regarding ethanol-mediated molecular cascades. Findings from the current review also support the ongoing use of IVM as a platform for developing new chemical entities that target P2X4Rs for the treatment of AUDs.

### Conflict of interest statement

The authors declare that the research was conducted in the absence of any commercial or financial relationships that could be construed as a potential conflict of interest.
